# QUALITY OF LIFE OF MOTHERS OF CHILDREN WITH CONGENITAL SYNDROME AFFECTED BY ZIKA VIRUS

**DOI:** 10.1590/1984-0462/2021/39/2019231

**Published:** 2020-08-21

**Authors:** Paulo Rogério Lobão de Araújo Costa, Francisca Bruna Arruda Aragão, Jacira do Nascimento Serra, Marcelo Sousa Andrade, Andréa Dias Reis, Maria do Desterro Soares Brandão Nascimento

**Affiliations:** a Universidade Federal do Maranhão, São Luís, MA, Brazil.; b Universidade de São Paulo, Ribeirão Preto, SP, Brazil.; c Universidade Estadual Paulista, Presidente Prudente, SP, Brazil.

**Keywords:** Quality of life, Microcephaly, Zika virus, Qualidade de vida, Microcefalia, Zika vírus

## Abstract

**Objective:**

To investigate how mothers of children with congenital syndrome (microcephaly) associated with Zika virus perceive their quality of life.

**Methods:**

This is a qualitative study carried out in two stages: at the Maternal and Child University Hospital and at Casa de Apoio Ninar through semi-structured interviews with mothers of children with congenital syndrome associated with Zika virus. Ten women comprised the sample.

**Results:**

In line with the critical discourse analysis, four categories emerged based on Fairclough’s 2008 assumptions: Quality of Life and Health - quality of life perception associated with the broad meaning of the term health; Quality of Life and Health Care Systems - characterized by care instability and fragmentation; Quality of Life and Free Time - lack of free time for activities of daily living and leisure; Quality of Life and Future Perspectives - lack of future perspective, considering that most participants had to stop working and studying to take care of their children.

**Conclusions:**

Mothers of children with congenital syndrome associated with Zika virus are devoted to their children’s care. Their lack of prospects was considered a consequence of the lack of time for activities of daily living and leisure.

## RESUMO

**Objetivo:**

Investigar a percepção da qualidade de vida das mães de crianças portadoras de síndrome congênita (microcefalia) associada ao Zika vírus.

**Métodos:**

Trata-se de um estudo qualitativo realizado em duas etapas. A primeira foi conduzida no Hospital Universitário Materno-Infantil e a segunda na Casa de Apoio Ninar, por meio de entrevista semiestruturada com mães de crianças que apresentam a síndrome congênita associada ao Zika vírus. A amostra foi composta por 10 mulheres.

**Resultados:**

Em consonância com a análise crítica do discurso, segundo os pressupostos de Fairclough, de 2008, quatro categorias emergiram: Qualidade de Vida e Saúde - a percepção da qualidade de vida está vinculada ao significado amplo do termo saúde; Redes de Qualidade de Vida e Atenção à Saúde - caracterizadas por precariedade e fragmentação do cuidado; Qualidade de Vida e Tempo Livre - falta de tempo livre para realizar atividades da vida diária e lazer; Qualidade de Vida e Perspectivas Futuras - foi abordada a falta de perspectiva futura, uma vez que a maioria das participantes teve que parar de trabalhar e estudar para cuidar dos filhos.

**Conclusões:**

Mães de crianças com síndrome congênita associada ao vírus Zika são totalmente dedicadas ao cuidado de seus filhos. A falta de perspectivas para o futuro foi considerada em decorrência da falta de tempo para lazer e atividade da vida diária.

## INTRODUCTION

The term quality of life (QoL) was used in Eastern and Western cultures. In ancient Chinese philosophy, it covers their art, literature, philosophy, and traditional medicine, as well as the positive and negative forces represented by Yin and Yang definitions. From the Western perspective, QoL was first related to the Aristotelian vision, which described happiness as a certain type of soul activity, such as feeling complete and fulfilled.^1^ We must adopt a perspective or a complex paradigm of the world to understand the area of knowledge in QoL better. The association between men, nature, and the environment surrounding them expresses it.^2^ Thus, we need to understand and study the repercussions of QoL and its association with changes in the daily life of mothers of children with congenital syndrome (microcephaly), as this concept is broad and may vary from individual to individual.^3^

Microcephaly is a congenital malformation in which the brain is not properly developed.^4^ Severe microcephaly occurs when the head circumference is more than three standard deviations below the average for age and gender.^5^ ^,^ ^6^ After the confirmation of Zika virus (ZV) in Brazil, hospitalizations increased due to several neurological manifestations. Descriptive studies conducted in the States of Pernambuco and Bahia identified that most patients with congenital syndrome had a clinical condition suggestive of arbovirus, presenting rash, arthralgia, and fever.^4^ ZV was established as the cause of microcephaly and other severe brain anomalies. The Department of Health of Pernambuco detected an unexpected increase in live births with microcephaly in October 2015, which caused a more direct communication between health authorities.^5^

Hence, this study aims to investigate how mothers of children with congenital syndrome (microcephaly) associated with ZV perceive their QoL.

## METHOD

This research was developed in two stages: at the Maternal and Child University Hospital of Universidade Federal do Maranhão (HUUFMA) and at Casa de Apoio Ninar. In the first stage, we conducted semi-structured interviews about registered cases of newborns diagnosed with congenital syndrome, following the guidelines of a booklet prepared by the Brazilian Ministry of Health, and about mothers with confirmed ZV infection, from November 2015 to May 2017. The second stage was in July 2018 and included mothers of children with congenital syndrome (microcephaly) associated with ZV, who had been hospitalized at the HUUFMA and had their children cared for at the studied institution.

A health professional at Casa de Apoio Ninar interviewed the participants at a convenient moment for them in order to create a space for dialog, knowledge, and construction, making them more comfortable to express their opinions. These items had been defined in the guidelines prepared by the Brazilian Ministry of Health.^7^

All mothers were first identified and invited by telephone to listen to a brief presentation of the research. The researcher was introduced, and an interview was scheduled to take place at the moment they would be at the institution.

Each participant received a questionnaire including close-ended questions about their sociodemographic characterization and a script with guiding questions to determine if mothers of children with congenital syndrome associated with ZV understood what QoL meant and the influence of this care on their QoL and activities of daily living. The interviewer transcribed the answers, which were later used for data analysis. The participants’ common statements were divided according to Fairclough’s assumptions.^3^ All mothers signed the Informed Consent Form. The Research Ethics Committee of UFMA approved this study (protocol No. 2,724,314).

We used a questionnaire (available with the corresponding author) including close-ended questions about the respondents’ sociodemographic characterization and a script with guiding questions to determine if mothers of children with congenital syndrome associated with ZV understood what QoL meant for them and the influence of this care on their QoL and activities of daily living.

Data were analyzed following the critical discourse analysis (CDA). The understanding of the world occurs through social processes, which are cultural and historical in turn. For Fairclough, discourse is a social and not purely individual practice. Therefore, the individual’s discourse is a consequence of situational variables.^3^ Thus, “discourses do not just reflect or represent social entities and relations, they construct or “constitute” them; different discourses constitute key entities [...] in different ways, and position people in different ways as social subjects.”^3^ Four categories were developed according to the CDA: QoL and Health, QoL and Health Care Systems, QoL and Free Time, and QoL and Future Perspectives. They were based on the answers of mothers’ discourses during the interviews.

## RESULTS AND DISCUSSION

Mothers treated in the day scheduled for the interview comprised the qualitative sample. The participants were named after flowers to protect their identity. Regarding the respondents’ sociodemographic aspects, nine of them did not have a formal job and were housekeepers, and only one had a formal job. As to their educational level, eight had a high school degree; one had an elementary school degree; and one had a higher education degree. The most common occupation was “housekeeper” (nine references). One participant had a higher education degree and worked as a social worker. Nine participants declared being brown-skinned and one, black-skinned (Table 1). All children were diagnosed with microcephaly and aged between zero and three years.

Four thematic categories emerged in line with the described method: QoL and Health, QoL and Health Care Systems, QoL and Free Time, and QoL and Future Perspectives.

**Table 1:** Sociodemographic data of participants.

Mother	Age (years)	Marital status	Schooling	Profession	Total of children	Total of individuals living in the same house
1	19	Single	High school	Housekeeper	1	4
2	34	Married	High school	Housekeeper	2	4
3	35	Married	High school	Housekeeper	3	6
4	32	Single	High school	Housekeeper	1	5
5	23	Single	High school	Housekeeper	2	5
6	19	Single	High school	Housekeeper	1	5
7	34	Married	Higher education	Social worker	1	3
8	34	Single	High school	Housekeeper	2	6
9	35	Married	Elementary school	Housekeeper	3	8
10	21	Single	High school	Housekeeper	2	4

### 1^st^ category: Quality of Life and Health

For the respondents, QoL is associated with a broad meaning of health care. The statements describe both physical health and QoL provided by health care networks of the Brazilian public health system (SUS, acronym in Portuguese):

(...) It means being healthy to be able to work, etc. - *Girassol*

(...) Being healthy, having a job, leisure time, and culture. - *Bromélia*

(...) Being healthy, having a house for me to live in, and taking care of my son. - *Tulipa*

(...) It is certainly being healthy, first, because it is everything. - *Lírio*

(...) Being healthy and financially stable and able to have fun. - *Margarida*

(...) It means, mainly, knowing how to take care of yourself, being emotionally well to be able to take care of another person. - *Hortênsia*

Pregnancy is a moment that represents social fulfillment in many cases. It is a symbol of the father’s masculinity and the mother’s emotional satisfaction. At this period, despite fear and anxiety, the couple fantasizes, makes plans, and wonders how their child will look. Thus, the life project of a child starts long before their birth by their parents. Plans are usually formulated for a child who has a perspective of “regular psychic and physical abilities.^”^ ^5^

The World Health Organization (WHO)^8^ defines health as “a state of complete physical, mental, and social well-being and not merely the absence of disease or infirmity.” The association between QoL and health was first seen in the 1980s and 1990s. Minayo^9^ mentions: “in the health field, the association between health and QoL, although quite unspecific and generalized, has existed since the birth of social medicine in the 18^th^ and 19^th^ centuries, when systematic investigations began to endorse this thesis and provide support for public policies and social movements.”^10^

The term QoL, when related to health, may be associated with socioeconomic, cultural, and personal experiences, as well as the lifestyle of each person.^11^ Concerns for the QoL concept in health are relatively new and partly stem from new paradigms that have influenced the policies and practices of services in recent decades.^12^

Health is a broad concept that includes several areas of social, economic, political, cultural, and human knowledge because it assumes a holistic idea of a person.^12^ “Health is a temporal notion that results in future experiences, which are determined by time and cannot be explained once and for all.”^13^ The health concept is dynamic and difficult to define and measure. Health is said to be a state, a QoL influenced by physical, mental, social, economic, and environmental factors.^14^

Health-related QoL is a subset of QoL aspects associated with one’s existence. According to the WHO, health is a dimension of our QoL.^8^ “A population’s measurement of health status allows defining levels of comparison between groups to detect inequities in health conditions according to different pathologies, geographical areas, economic conditions, gender, or age.”^9^ Thus, QoL assessment becomes a part of clinical practice in order to measure problems that interfere with the patients’ well-being and life, constituting effective measures for the therapeutic evaluation of patients and groups of patients.^15^

The respondents associated the QoL notion with an extensive meaning of the health term. Therefore, their statements describe not only physical health but also the quality of health care services provided by federal, state, and municipal organizations and institutions of direct and indirect administration and foundations maintained by the Brazilian public power.

(...) Being respected as a person, with proper and well-equipped healthcare services and hospitals; right to equal education, a decent job, and respect, as well as leisure time. - *Orquídea*

(...) Proper home, food, education, and great health. - *Violeta*

### 2^nd^ category: Quality of Life and Health Care Networks

The association between health care networks and QoL addressed by the study participants has been described in arrangements that contribute to meet the needs of comprehensive health services and quality for the assisted population. Primary care as the main factor for the maintenance of health care networks becomes important in the discussion about health problems that require greater care.^16^

The healthcare process for children with chronic conditions results in a lot of difficulties for families concerning their social interaction, physical and emotional burden, as well as frequent treatments at outpatient and rehabilitation services, due to emerging complications. The childcare routine causes changes in the family, especially for the mother because she is the one who most often undertakes the mission of taking care of her child.^17^

The right to health - apart from being fundamental to everyone - represents an inseparable constitutional consequence of the right to life.^18^ “Therefore, primary health care should be the user’s gateway to the health system, as it provides the population with access to integrated health networks.”^16^

The services for the population must follow the principles of SUS to guarantee the rights of the patient, regardless of gender, ethnicity, occupation, or other social or personal characteristics. Care should aim for a continuous service, which depends on the ability to ensure the flow of information.^19^

Primary care is an approach that forms the basis and determines the work of all other health system levels. It addresses the most common community problems, offering preventive, healing, and rehabilitation services to maximize health and well-being.^20^ Care organizes and rationalizes the use of both basic and specialized resources aimed at promoting, maintaining, and improving health.^19^

In addition to health care instability for pregnant women, the participants’ declarations showed that child care is also compromised, given the great delay in the service and scheduling of appointments:

(...) Today, when you go to an appointment, the doctor requests exams, then you wait months in the queue to be able to take them, and when you manage to take them, you have to wait even longer to return to the doctor [...] all this complicates even more my son’s situation. - *Orquídea*

Based on this discussion about health care networks and their influence on the QoL of the study participants, we found that public health policies should be improved for better effectiveness and opportunity for the population.

A national reform oriented toward connecting economic factors and social needs is necessary to create a stable and continuous process of social inclusion, and, above all, to establish health policies as State policies.^18^

The reformulation of health policies as an attempt to improve public health indicators has been addressed. Therefore, we need coordinated and coherent interventions to improve the health situation and reduce inequities, considering this approach for social determinants. In turn, good health contributes to other social priorities, such as well-being, education, social cohesion, environment preservation, increased productivity, and economic development. This scenario creates a “virtuous circle,” in which health and its determinants provide feedback and benefit to each other.^21^

### 3^rd^ category: Quality of Life and Free Time

When participants were questioned about their daily routine, lack of free time to perform activities of daily living was the most mentioned variable, considering that a great part of their time is dedicated to taking care of their children and constant visits to health services:

(...) It is basically just taking care of my son. - *Hortênsia*

(...) It changed a lot because ever since she was born, my time has been dedicated to her. I do not have time, not even to take care of myself. - *Margarida*

(...) Only taking care of my son and taking him to the doctor. - *Tulipa*

(...) It changed the routine of having time to do everything. I have almost daily appointments, physical therapy, occupational therapy, speech therapy. But it is okay. - *Violeta*

(...) My life boils down to daily appointments, no time to sleep properly or for leisure. - *Rosa*

The long-term consequences of microcephaly depend on the underlying brain abnormalities and may range from mild delays in intellectual development to motor deficit, such as cerebral palsy.^22^ Children with microcephaly require full assistance from those involved in the care process, due to the limitations of this disease on their development. This fact interferes with the experience of caregivers in social, emotional, self-care, and professional fields.^23^

Mothers are the main affected caregivers. The caregiver should be constantly attentive and always ready and available to assist the child, who demands care related to hygiene, food, and particularly the prevention of accidents.^23^

### 4^th^ category: Quality of Life and Future Perspectives

QoL at work is considered the satisfaction of the employee’s demands. Once met, it is associated with other variables, which include organizational commitment, job satisfaction, and fulfillment in different life domains, such as family, leisure, health, education, friendship, culture, social status, among others:^23^

(...) It is not good because I cannot work anymore. In addition, I have other stuff, like a daughter (sister), who became ill after the birth of my son with microcephaly. So, I have one daughter at follow-up and another one being treated for transverse myelitis. - *Orquídea*

(...) Due to the total dedication to my son, I do not have much time to work and take care of my QoL. - *Bromélia*

(...) It has changed a lot of my life routine because I had to stop working and studying. Today, I only receive the Continuous Provision Grant from the government. - *Jasmim*

Studies on ZV-related microcephaly have reported that mothers and their children with this malformation are prone to a life of great difficulty, due to the child’s condition.^23^ Another issue listed by caregivers is the increase in expenses caused by this disease, such as: appointments, medication, transport, food, among others. Several families depend on benefits provided by the State as the single household income.^23^

Hence, the QoL of participants who quit their jobs to take care of their children is associated with a lack of satisfaction in other life domains, such as family, leisure, health, education, friendship, culture, and social status, influencing their activities of daily living. The participants addressed their lack of future perspectives, given that most of them had to stop working to take care of their children.

This study has several limitations, including the external validity of the findings inherent to the design. Also, we highlight the lack of child characterization, which we suggest for further studies. Nonetheless, our strength was the analysis of mothers who had children with microcephaly, regardless of the level of impairment of the child

In conclusion, the interviewed mothers of children with congenital syndrome associated with ZV were fully devoted to their children’s care. The impact of this care on the participants’ QoL was evidenced by their lack of prospects as a consequence of lack of time for activities of daily living and leisure.
